# Impact of tumor heterogeneity on disease-free survival in a series of 368 patients treated for a breast cancer

**DOI:** 10.1186/1746-1596-8-S1-S43

**Published:** 2013-09-30

**Authors:** Myriam Oger, Mohamed Allaoui, Nicolas Elie, Jacques Marnay, Paulette Herlin, Benoît Plancoulaine, Jacques Chasle, Véronique Becette, Catherine Bor-Angelier

**Affiliations:** 1 Imagin' Team of EA 4656 in François Baclesse Cancer Centre, 3 avenue du Général Harris, 14076 Caen, France; 2 Pathology laboratory, François Baclesse Cancer Centre, 14076 Caen, France; 3 Plateau d’Histo-Imagerie Quantitative, SF ICORE, CMABIO, University of Caen Basse-Normandie, 14076 Caen, France

## Introduction

Tumor heterogeneity [1-4] is an old concept but its impact on the cancerogenesis process is poorly understood. Breast cancer is a noteworthy model for its frequency, and for the diversity of its phenotypes and of its evolution. This study examines the influence of the heterogeneity of tumor proliferation on disease-free survival of patients with a breast carcinoma.

## Material and methods

### Histological slides

The study involved a series of 368 patients from the François Baclesse Cancer Centre (Caen) treated for a breast carcinoma between 1991 and 1995, whitout neoadjuvant therapy and with a follow-up of more than 15 years. The table 1 contains the description of the series.

**Table 1:** Univariate Analysis of Disease Free Survival – 368 Eligible Patients. In grey: Follow up (2011)

Variable		No. of patients	*P *value
Age			
	Mean = 58.7 yr		0.400
Menopauses			
	Yes	237 (65%)	
	No	124 (34%)	
Localization			
	Right breast	176 (47.8%)	
	Left breast	187 (50.8%)	
	Synchronous bilateral	5 (01.4%)	
Tumor size			
	Mean = 25 mm		0.010
Surgery			
	Tumorectomy	212 (57.6%)	
	Mastectomy	141 (38.3%)	
	Biopsy	15 (04.1%)	
Excision quality			
	Satisfying	202 (57.2%)	
	Unsatisfying	47 (13.3%)	
	Unspecified	104 (29.5%)	
Histological type			
	ICC	297 (80.71%)	
	ILC	37 (10.05%)	
SBR grade			
	Grade 1	50 (14%)	
	Grade 2	180 (49%)	0.002
	Grade 3	137 (37%)	
Mitotic index (/1.7mm²)			
	Mean = 10 mitosis		<0.0001
Tumor vascular emboli			
	Yes	293 (79.6%)	
	No	75 (20.4%)	
Lymph node metastasis			
	Yes	153 (44%)	<0.0001
	No	195 (56%)	
Hormone receptor status (at least 1)			
	Yes	265 (73%)	0.030
	No	98 (27%)	

Visceral or lymph node (other than axillary) metastasis			
	Yes	136 (37%)	
	No	232 (63%)	
Local recurrence			
	Yes	66 (18%)	
	No	302 (82%)	
Oncological event (Metastasis and/or local recurrence)			
	Yes	157 (42.5%)	
	No	211 (57.5%)	
Death			
	Yes	90 (24.46%)	
	No	278 (75.54%)	

**Figure 1:**
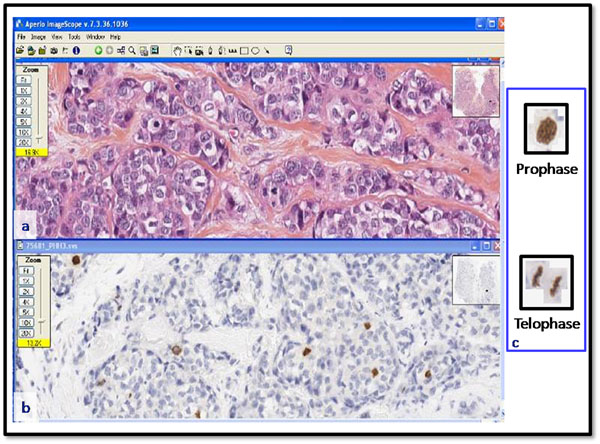
**dyes used to stain the histological sections. a**: histological slide stained with HES; **b**: histological slide of the same case immunohistochemically stained with PHH3 [6-8] (anti-phosphohistone-H3 antibody); **c**: thanks to this specific immunohistochemical, identification of cells with mitosis figures, from prophase to telophase, is improved.

Histological sections, representative of each tumor, have been stained with the anti-phosphohistone-H3 antibody (PHH3: Ser10, MILLIPORE®, dilution 1/600) [5,6]. With this specific immuno-stain, cells presenting mitosis figures are more easily identifiable (Figure 1).

### Acquisition

Histological slides have been scanned with a high resolution slide scanner to obtain virtual slides with a final resolution of 0.5 µm (ScanScope® CS from Aperio Technologies (20x NA 0.7 objective). The true color images obtained (color RGB 24 bits) have been saved in the tiled pyramidal TIFF file format.

### Region of interest (ROI)

Before the automatic image analysis, the user can discard “normal” tissue surrounding the tumor by drawing a region of interest on the high resolution virtual slide with the Aperio ImageScope® software.

### Image processing

The image processing was performed in two steps on a personal computer with a 1.6 GHz Pentium IV processor and a 1 GB of random access memory (RAM). The first step being a sub-sampling of virtual slide done with a specific algorithm 'Daubechies' second moment orthogonal wavelet decimation developed in C++ language which creates a low resolution image of the virtual slide (divided by 8: from 0.5µm to 4µm/pixels). In a second step, the low resolution image is automatically processed thanks to chaining operators of image analysis toolbox software (Aphelion, ADCIS).

In addition to estimating the frequency of mitotic figures, the program detects “hot spots” and measures 9 features representing the tumor heterogeneity, including the Haralick texture features and Fisher’s index. The zones of influence of each stained nuclei have been determined using Voronoï’s pavement principle. When nuclei are close, the size of pavements is small, highlighting the “hot spots”.

### Feature selection

A principal component analysis has been done in order to select the most relevant features.

### Statistic analysis

These features have been statistically analyzed, combined with classic clinic-pathological prognostic factors (age, tumor size, grading, mitotic index, vascular emboli and metastatic lymph nodes).

## Results

### Principal component analysis

Thanks to the principal component analysis (PCA) 4 features representing tumor heterogeneity have been chosen then combined into three new features: CP1, CP2 and CP3, corresponding to the three principal directions of the PCA.

The four selected features are:

- 2 Haralick’s texture indexes (correlation and energy);

- Fisher’s index;

- variance of the size of Voronoï pavements.

The variance of the size of Voronoï pavement (named Voronoï) and the Fisher’s index are regional features whereas the Haralick’s texture indexes are local features. Indeed, Voronoï and Fisher features are “cutting” the tissue into pieces and analyzing each of them compared to the others, whereas Haralick is dealing with relations between neighbor pixels, each pixel representing a cell at this resolution.

### Prognostic study

In the analysis of prognostic factors, disease free survival was used as the end point.

#### Univariate statistical analysis (DFS)

Univariate analysis of disease free survival was performed with the features of age, tumor location, initial tumor size, pathologic lymph node status (N), histological type, SBR grade, mitotic index, vascular emboli, metastatic lymph nodes and hormone receptor status. The results are shown in Table 1 for usual features, in Table 2 for heterogeneity features.

**Table 2:** Results of the univariate analysis

Variables	P value
Voronoï	0.040

Normalized variance of density	0.170

Energy (Haralick)	0.090

CP1	0.500

CP2	0.016

CP3	0.670

The CP2 feature correlated highly with disease free survival, whereas the variance of the Voronoï pavements was borderline significant.

#### Multivariate statistical analysis (Cox)

The above features that correlated with disease free survival in univariate analysis were combined with clinic-pathologic factors and included in the multivariate analysis. Cox’s regression analysis highlighted 3 independent prognostic factors: tumor heterogeneity feature CP2 (RR = 1.46; p = 0.03), mitotic index (RR = 1.71; p = 0.004) and lymph node metastasis (RR = 2.20, p < 0.0001) correlated highly with disease free survival.

The construction of this model has individualized 3 groups of patients: 0 factor, 1 or 2 factors and 3 poor prognostic factors (mitotic index > 10, lymph node metastasis in the axillary dissection, upper tercile of CP2; p < 0.0001).

**Figure 2:**
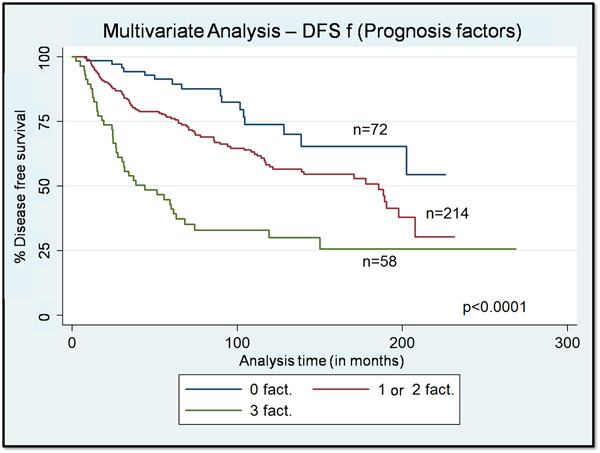
**Model build using the results of the multivariate analysis.** Multivariate analysis highlighted 3 independent prognostic factors: tumor heterogeneity (CP2), mitotic index, lymph node metastasis. The construction of this model has individualized 3 groups of patients: 0 factor, 1 or 2 factors and 3 poor prognostic factors (mitotic index > 10, lymph node metastasis in the axillary dissection, upper tercile of CP2; p < 0.0001).

Disease free survival according to this model is shown in Figure 2.

## Discussion and conclusion

To characterize tumor heterogeneity in the presented series of breast cancer, 9 features were computed. 4 nonredundant of them have been selected by principal component analysis (PCA).

PCA was also used to create 3 new composite features: CP1, CP2 and CP3, corresponding to the 3 principal directions of the PCA.

The univariate analysis made for each feature from image analysis has first highlighted that only the combination CP2 and Voronoï’s feature had a prognostic value. It has to be noted that a high value of heterogeneity index is associated with a poor prognosis.

In multivariate analysis, CP2 was found to be an independent prognostic feature just like the mitotic index and the lymph node status. The lymph node status is a well-known clinical factor; the two other features are intrinsic factors of tumor growth, at cellular level for mitotic index and at the tissue level for heterogeneity.

Surprisingly, age, tumor size, Scarff and Bloom Grade and hormone receptor status are of secondary importance compared to these 3 features.

This result encourages to confront the heterogeneity feature CP2 to clinic information, such as recent or late oncologic event or the nature locoregional or distant visceral of the recurrence, and to the absence of lymph node metastasis.

## Competing interests

The authors declare no competing interests.

## Authors' contributions

MO, MA, NE, JM, PH, BP and CB defined the research theme, analyzed the data and interpreted the results. NE, PH and BP designed and carried out experiments for the computation the heterogeneity features. JC, VB and CB collected the data for classical clinic-pathological prognosis factors such as histological type and grade of the tumor, size or mitotic index . VB drawn the Regions of Interest for all the images. MO carried out the experiments to select the most relevant heterogeneity features and create the new CP2 feature. JM carried out all the statistical analyses. MO and MA wrote the paper.

All authors read and approved the final manuscript.
